# Diagnostic stewardship to improve patient outcomes and healthcare-associated infection (HAI) metrics

**DOI:** 10.1017/ice.2023.284

**Published:** 2024-04

**Authors:** Harjot K. Singh, Kimberly C. Claeys, Sonali D. Advani, Yolanda J. Ballam, Jessica Penney, Kirsten M. Schutte, Christopher Baliga, Aaron M. Milstone, Mary K. Hayden, Daniel J. Morgan, Daniel J. Diekema

**Affiliations:** 1 Division of Infectious Diseases, Weill Cornell Medicine, New York City, New York; 2 Practice, Sciences, and Health Outcomes Research, University of Maryland School of Pharmacy, Baltimore, Maryland; 3 Department of Medicine–Infectious Diseases, Duke University School of Medicine, Durham, North Carolina; 4 Infection Prevention and Control, Children’s Mercy Kansas City, Missouri; 5 Division of Geographic Medicine and Infectious Diseases, Tufts Medical Center, Boston, Massachusetts; 6 Medical Director, Infectious Disease, eviCore Healthcare, Bluffton, South Carolina; 7 Section of Infectious Diseases, Department of Medicine, Virginia Mason Hospital and Seattle Medical Center, Seattle, Washington; 8 Division of Pediatric Infectious Diseases, Johns Hopkins Medicine, Baltimore, Maryland; 9 Division of Infectious Diseases, Rush University Medical Center, Chicago, Illinois; 10 Department of Epidemiology and Public Health, University of Maryland School of Medicine, Baltimore, Maryland; 11 Veterans’ Affairs Maryland Healthcare System, Baltimore, Maryland; 12 Division of Infectious Diseases, Department of Internal Medicine, University of Iowa Carver College of Medicine, Iowa City, Iowa; 13 Division of Infectious Diseases, Department of Medicine, Maine Medical Center, Portland, Maine

## Abstract

Diagnostic stewardship seeks to improve ordering, collection, performance, and reporting of tests. Test results play an important role in reportable HAIs. The inclusion of HAIs in public reporting and pay for performance programs has highlighted the value of diagnostic stewardship as part of infection prevention initiatives. Inappropriate testing should be discouraged, and approaches that seek to alter testing solely to impact a reportable metric should be avoided. HAI definitions should be further adapted to new testing technologies, with focus on actionable and clinically relevant test results that will improve patient care.

Healthcare-associated infections (HAIs) are common causes of morbidity and mortality.^1^ Emphasis on quality metrics across hospitals and financial incentives for hospitals to reduce HAI rates is increasing. Diagnostic testing plays a key role in the detection of HAIs and reportable events. Diagnostic stewardship can be leveraged to increase appropriate testing, decrease inappropriate testing, and in turn improve the accuracy of HAI diagnosis. As a result, hospital-based quality initiatives and infection prevention programs should include diagnostic stewardship initiatives to reduce misclassification of colonization or contamination events as HAIs. Diagnostic stewardship refers to the process of modifying the ordering, collection, performance and/or reporting of diagnostic tests to improve the diagnosis of and treatment of infections and other conditions.^2^ The principles of diagnostic stewardship^3^ and related issues have been outlined in a series of publications by the SHEA Diagnostic Task Force.^3–5^ Here, we review the interplay between HAIs and diagnostic stewardship. Table 1 lists examples of diagnostic strategies for HAIs.

**Table 1.** Examples of Diagnostic Stewardship Strategies for NHSN-Reportable HAI

CAUTI	**Ordering** Require guideline-supported indications for urine cultures. Educate on prevalence of asymptomatic bacteriuria in catheterized patients. **Collection** Advise replacing catheter prior to culture if in place >7 days. Do not collect urine sample from urine collection bag. Reduce delays in transport, refrigerate if >1 hour delay, use collection. device that contains a preservative (eg, boric acid). **Performance** Reduce colonization detection by performing reflex testing (eg, culture only if pyuria present). **Reporting** Include nudges advising against treatment of asymptomatic bacteriuria.
HO-CDI	**Ordering** Limit testing of patients receiving laxatives. Limit repeat tests within given period. Do not test patients without symptoms of CDI. **Collection** Reduce collections received >24 hours after initial orders. **Performance** Do not test formed stool (Bristol stools<5, or negative stick test).^50,51^ Use algorithm that includes both NAAT and toxin EIA if NAAT+. **Reporting** Include nudge that a positive NAAT test could represent colonization in the absence of clinical disease and detection of colonization is not indication for treatment. Consider Infectious disease consultation if uncertain.
CLABSI	**Ordering** Avoid blood cultures in patients with low probability for bacteremia with the use of CDS and education. Eliminate surveillance blood cultures. Develop guidance to standardize indications for blood cultures. **Collection** Optimize technique (skin prep, volume, and number of cultures). Avoid drawing blood cultures through central lines. **Performance** Implement rapid diagnostics. **Reporting** Include nudge about common commensals/likely contaminants.

Note. NHSN, National Healthcare Safety Network; HAI, healthcare-associated infection; CAUTI, catheter-associated urinary tract infection; HO-CDI, hospital-onset *Clostridioides difficile* infection; CLABSI, central-line–associated bloodstream infection; CDS, clinical decision support; NAAT, nucleic acid amplification test; EIA, enzyme immunoassay.

**Table 2.** Examples of the Impact of Diagnostic Stewardship Interventions on Patient and HAI Outcomes

	CAUTIs				
	Author/Year	Intervention	Antibiotics, %	Cultures, %	Reported HAIs, %
Ordering	Keller 2018^52^	BPA to avoid cultures for ASB	↓ 0.5	↓ 6.3	…
	Trautner 2015^53^	Appropriate testing algorithm, clinical detailing, peer-to-peer feedback	↓ 35*	↓ 53*	↓ 1.5
	Mullin 2017^29^	Improved electronic documentation of catheters, nurse protocols, indication education	…	…	↓ 37*
	Krouss 2023^54^	Educational BPA on high rates of ASB and indication requirements	…	↓ 20.9	↓ 21.6
Processing	Lynch 2020^35^	Conditional urine reflex culturing	…	↓ 39*	↓ 29*
	Sarg 2016^55^	Conditional urine reflex culturing with IUC	↓ 18^a^	↓ 30	…
	Claeys 2021	Conditional urine reflex culturing	…	↓ 21*	…

	HO-CDI				
	Author/Year	Intervention	Antibiotics, %	NAATs, %	Reported HAIs, %
Ordering	Shallal 2023^56^	CDS with hard stop for previous laxative use	--	↓ 28	↓ 57
	Solanky 2021^57^	Multi-step; room cleaning, hand-hygiene, FQ restriction, testing algorithm	NC	…	↓ 62*
	Madden 2019^15^	CDS and financial incentives	…	↓41*	↓ 31*
	Mizusawa 2019^58^	Soft & hard stop CDS based on laxative use and previous testing	↓ 31*	↓ 37*	…
Processing	Bork 2019^59^	Multi-step CDI testing algorithm	↓43*	NC	↓ 28*
	Markley^60^ 2020	Two-step CDI testing	…	NC	↓ 87*

	CLABSI				
	Author/Year	Intervention	Antibiotics, %	Cultures, %	Reported HAIs, %
Ordering	Woods-Hill 2017^43^	Fever checklist and blood culture ordering algorithm	…	↓ 46*	…
	Fabre 2020^47^	Blood culture algorithm, education, provider feedback	…	↓ 18 ICU* ↓ 30 floor*	…
	Woods Hill 2022^44^	CDS tools	↓ 13	↓33	↓36

Note. ASB, asymptomatic bacteriuria; BPA, best-practice alert; CDI, *C. difficile* infection; CDS, clinical decision support; CAUTI, catheter-associated urinary tract infection; HO-CDI, hospital-onset *Clostridioides difficile* infection; CLABSI, central-line–associated bloodstream infection; FQ, fluoroquinolone; HAI, healthcare-associated infections; ICU, intensive care unit; IUC, internal urinary catheter, NC, no change.

* Indicates *P* < .05.

a New antibiotics initiated in response to urine culture in the patient-level analysis.

The Centers for Disease Control and Prevention (CDC) National Healthcare Safety Network (NHSN) tracks HAIs in the United States. Currently, 5 HAIs are publicly reported: hospital-onset methicillin-resistant *Staphylococcus aureus* bacteremia (HO-MRSA), central-line–associated bloodstream infection (CLABSI), catheter-associated urinary tract infection (CAUTI), hospital-onset *Clostridioides difficile* infection (HO-CDI), and surgical-site infection (SSI). Among them, HO-MRSA bacteremia, CLABSI, CAUTI, and HO-CDI events require a positive test from the clinical laboratory to meet the HAI definition. However, a positive urine culture or test for *C. difficile* does not distinguish colonization or contamination from infection. Thus, testing practices can have a major impact on HAI rates, and the pressure to reduce HAIs has become an important driver of diagnostic stewardship.^4,5^ Here, we focus on the application of diagnostic stewardship interventions for CAUTI, HO-CDI, and CLABSI.

One of the main federal initiatives driving quality improvement, including HAI reduction, is financial incentives. In 2016, the Center for Medicaid and Medicare Services (CMS) began to link hospital payments to improvements in several quality measures.^6,7^ As these financial incentives threatened healthcare systems’ financial performance, Goodhart’s law began to apply: “When a measure becomes a target, it ceases to be a good measure.” Although the incentives were associated with reduced publicly reported HAIs,^8^ these penalties translated into millions of dollars of lost revenue for many hospitals.^9^ As a result of these pressures, some hospitals modified testing practices as a strategy to reduce HAI detection, through absolute reductions in testing without diagnostic stewardship (ie, “don’t look”) or through strategic reductions in inappropriate testing using diagnostic stewardship.^10,11^

We investigated the influence of diagnostic stewardship interventions on HAI prevention initiatives and the impact of these on HAI rates (summarized in Table 2). We reviewed the evidence for CAUTI, HO-CDI, and CLABSI diagnostic strategies aimed at improving patient care (patient-centered). In each HAI section, we have contrasted the patient-centered approach with an HAI metric-centered approach. The metric-centered approach focuses on achieving an absolute reduction in HAI rates through alterations in diagnostic testing instead of focusing on a patient-centered approach to reduce inappropriate testing using diagnostic stewardship. In the discussion, we have explored challenges and opportunities to leverage diagnostic stewardship for HAI reduction that maintains its focus on patient outcomes.

## *Clostridioides difficile* infection (CDI)

Increasing attention to HO-CDI has led to numerous changes in diagnostic testing over the past 20 years. Initial enzyme immunoassays (EIAs) to detect toxin had poor sensitivity, which ushered in the widespread use of nucleic acid amplification tests (NAATs) that detect toxin genes.^12^ The increased sensitivity of the NAAT platform led to increased detection of *C. difficile* (colonization and infection) and increased hospital-onset *C. difficile* rates due to overdiagnosis caused by misclassification of colonization as infection. Polage et al^13^ performed a prospective observational study of 1,416 adult patients comparing outcomes of NAAT and toxin EIA tests. Virtually all CDI complications and death occurred among patients with positive NAAT and positive toxin tests. However, patients with positive NAATs and negative toxin tests had outcomes similar to those without CDI, suggesting that exclusive reliance on NAAT tests results in HO-CDI overdiagnosis, overtreatment, and increased healthcare costs.^13^ In another retrospective study, Theiss et al^14^ compared detection of *C. difficile* with different testing algorithms. They compared glutamate dehydrogenase (GDH) testing with toxin testing of all positive results versus NAAT alone, and none of these testing approaches could adequately discriminate between colonization and infection.^14^

Although determining colonization versus infection can be challenging because of the need for clinical evaluation, optimizing the ordering and collecting steps is an important diagnostic strategy. Madden et al^15^ evaluated electronic clinical decision support (CDS) to provide guidance on ordering and collection combined with financial incentives to the ordering trainees to improve appropriate *C. difficile* testing. The CDS included notification of testing within the prior 28 days and practice guidelines, and it highlighted specific risk factors for HO-CDI: antibiotic use, intraabdominal surgery, and advanced age.^15^ This diagnostic stewardship intervention reduced *C. difficile* testing by 42% by reducing inappropriate testing. This reduction was sustained for at least 1 year. In addition to reducing testing, the intervention reduced HO-CDI reportable cases and resulted in financial savings.^15^ Similarly, a 15-hospital pragmatic intervention study created CDS to reduce duplicate *C. difficile* testing and testing in those who had recently received laxatives. This intervention reduced testing by 25%, oral vancomycin use by 15%–27%, and HO-CDI events by 31%–58%.^16^ Although most studies are conducted among an adult population, there are similar HAI diagnostic challenges among pediatric populations. The nature of their stool consistency and high infant colonization add to the difficulty of accurate diagnosis, as summarized by Sammons.^17^ Recent studies in children have shown that diagnostic stewardship addressing the ordering stage using CDS can reduce inappropriate *C. difficile* testing and observed HO-CDI cases.^18,19^

To help account for the increased sensitivity of NAATs,^20^ the CDC updated the LabID-event surveillance definition by basing the metric on the last test result in a HO-CDI multistep testing algorithm. For example, if an initial NAAT is positive but a subsequent toxin assay is negative, this would not be counted as a HO-CDI LabID-event. These revisions have partially addressed some of the concerns around the NHSN LabID-event definition,^21,22^ as well as providing incentive for healthcare facilities to implement 2-step testing and diagnostic stewardship. This adaptation of a national surveillance definition may reduce reported HO-CDI rates by >40% independent of any change in infection prevention practice or actual *C. difficile* infections.^23,24^

Although there is progress in improving the HO-CDI metric with patient-centered diagnostic stewardship strategies, metric-centered mitigation strategies that bypass stewardship may be occurring. Absolute reductions in tests for *C. difficile* combined with empiric treatment of any healthcare-associated diarrhea or screening every patient on admission (with subsequent empiric treatment if the patient develops symptoms) would reduce HO-CDI cases reported to NHSN but could harm patients. The potential unintended consequences of undertesting include missed diagnosis, treatment, and isolation to reduce the risk of nosocomial transmission, as well as over treatment of patients with non-CDI causes of diarrhea. Overtesting results in treatment of patients who are colonized without infection.^25,26^ Although it is unknown whether undertesting or screening on admission occurs, the CMS and the CDC jointly published a notice in 2015 about anecdotal reports of “systematic underuse or overuse of diagnostic microbiology testing” to avoid HAI reporting, cautioning against these approaches.^27^

Some diagnostic stewardship approaches to reduce HO-CDI rates have been successful; however, additional opportunities remain, including further changes in the surveillance definition. The CDC has proposed another surveillance definition revision (HOT-CDI)^28^ that incorporates treatment, to discriminate better between findings of colonization and clinically important infection. The impact of these changes on HO-CDI rates remains to be seen.

## Catheter-associated urinary tract infection (CAUTI)

Inappropriate urine testing for fever, delirium, and other nonspecific constitutional symptoms in hospitalized patients with urinary catheters has led to significant misclassification of colonization (catheter-associated asymptomatic bacteriuria) as infection, which can lead to overdiagnosis of CAUTI. Several diagnostic stewardship interventions have successfully decreased overdiagnosis of CAUTI. Mullin et al^29^ took a 6-pronged approach that combined ordering and collection guidance. Together, these interventions reduced inappropriate urine culture orders by 50% and CAUTI diagnoses by 33%. Several other studies have examined interventions to improve urine culture stewardship overall, for both catheterized and noncatheterized patients. Best-practice alerts reduced inappropriate ordering of urinalyses and urine cultures and resulted in less antibiotic prescribing.^30,31^ Another approach is to address laboratory processing through conditional urine reflex testing, wherein urine cultures are only processed if they meet prespecified criteria on urinalysis (UA) or urine microscopic examination. Several researchers have examined different screening cutoffs for number of white blood cells per high-powered field (WBC/hpf), and although there is no single consensus for all populations,^32,33^ >10 WBC/hpf is most common.^34^ Lynch et al^35^ introduced a system-based approach of conditional reflex urine cultures in a VA hospital and found a 38% decline in urine culturing in acute-care settings, a 39% decline in the emergency department, and 89% reduction in long-term care centers.^35^ Claeys et al^33^ also found that conditional reflex urine culturing resulted in a 21% relative reduction in urine cultures in 3 Veterans’ Affairs hospitals that had such policies compared to 3 VA hospitals that did not. Notably, they found no harms, such as increase in secondary bacteremia among the hospitals with the new policies.^33^ Daley et al^36^ modified reporting by requiring all providers to call the microbiology laboratory for urine-culture results, which translated into a large reduction in inappropriate prescribing with no negative consequences.^36^ In contrast to adult populations, pediatric UTI diagnosis is more complex and varies by age. Only limited data currently inform urinary diagnostic stewardship. In a recent study in children, CDS increased urinalysis collection by 23% and reduced urine culture use by 36%.^37^ Additional studies are needed in this area.

Blanket reductions in urine culturing without concern for clinical appropriateness could be harmful. Although it is difficult to quantify the frequency of these occurrence, Ider et al^39^ and Horowitz et al^38^ have summarized these concerns in qualitative studies. Examples of potentially harmful practices include delaying urine-culture collection until a catheter is out for 48 hours (avoiding attribution to the catheter), delaying cultures until the patient is on appropriate antibiotics (more likely to be culture negative), culturing all patients with catheters on admission (avoiding CAUTI attribution), and treating empirically without collecting a urine culture (avoiding cases). Although these approaches could lower CAUTI rates, they could harm patients through increased antibiotic use, promoting antimicrobial resistance, and delaying diagnosis. Therefore, it is essential that an adjustment in testing practices occur in conjunction with diagnostic stewardship interventions that target inappropriate cultures, not all cultures.

Similar to HO-CDI, the CDC has revised the CAUTI definition in an attempt to discriminate better between clinical infection and colonization. In 2015, the definition was updated to remove urinalysis criteria, increase the urine-culture bacterial quantity threshold, and exclude yeasts or molds as CAUTI pathogens.^40^ This definition update to include specimen processing resulted in a >40% decline in reportable CAUTIs.^41^

In summary, patient-centered diagnostic stewardship, which focuses on optimizing urine cultures, has led to less antibiotic use and reduced reportable CAUTI rates while avoiding the potential harms of indiscriminate reductions in urine culturing. Adapted surveillance definitions can help focus on events that contribute to patient harm and should be the target of ongoing diagnostic stewardship and infection prevention initiatives.

## Central-line–associated bloodstream infection (CLABSI)

Inappropriate ordering and collecting of blood cultures remains a driver of high blood-culture contamination and CLABSI rates. CLABSI reduction is a major target of infection prevention and quality and safety groups through optimizing use of central venous catheters and diagnostic stewardship. Blood-culture ordering and collection practices can have an impact on CLABSI rates. Niedner et al^42^ surveyed 16 pediatric intensive care units (PICUs) at 14 hospitals, finding that CLABSI rates correlated significantly with the “aggressiveness score” of their blood-culture ordering and collection practices, which included practices such as taking samples from multiple lumens of a central venous catheter.^42^ Woods-Hill et al^43^ performed a study in children and found that improving blood-culture ordering could safely reduce culture rates. This research led to development of consensus recommendations for ordering blood cultures in critically ill children and to a multicenter diagnostic stewardship study in 14 PICUs. These interventions led to reduced blood-culture rates (33%), broad-spectrum antibiotic use (13%), and CLABSI rates (36%), without harms.^44,45^ Noting a similar gap in evidence-based guidance for ordering blood cultures in hospitalized adults, Fabre et al^46^ reviewed ordering practices and devised an algorithm to improve appropriate ordering. In 2020, electronic CDS plus education on when to draw blood cultures reduced inappropriate testing without any negative effects on sepsis or mortality.^47^

Other challenges with CLABSI are technological advances in the detection of organisms causing bloodstream infection using molecular and other non–culture-based tests (NCTs).^48^ There is concern that CLABSI reporting could become a barrier to adoption of more sensitive diagnostics, which otherwise hold promise for more rapid and accurate detection of BSI. To address this concern, the NHSN CLABSI definition has been adjusted so if a non–culture-based test identifies a pathogen, but a blood culture drawn within 2 days before or 1 day after the non–culture-based test is negative, only the results of the blood culture will be used to make a BSI determination.^40^ This allows the laboratory to introduce non–culture-based tests that are more sensitive without changing NHSN CLABSI rates, provided they continue to perform blood cultures in parallel. The final value of non–culture-based tests remains to be determined. Although non–culture-based tests may have higher sensitivity, they cannot always discriminate true infection from contamination or from detection of nonviable organisms. As diagnostic technology improves, it is important that pressure to reduce HAI rates does not prevent laboratories from adopting advances that otherwise improve patient care.^10^

Although patient-centered diagnostic stewardship of blood culturing is the best approach to patient care, potential metric-focused strategies that bypass diagnostic stewardship, such as avoiding blood cultures in favor of empiric treatments, could reduce CLABSI rates but are not recommended because of the potential for patient harm. The harms associated with these mostly theoretical testing practices are similar to those for other HAIs: delayed diagnoses, unnecessary treatment, adverse events, antimicrobial resistance, and undetected pathogen transmission.

Like CAUTI and HO-CDI, the CLABSI definition presents opportunities for improvement. The CDC NHSN plans to introduce a new surveillance definition that may eventually replace or complement CLABSI. This metric is hospital-onset bloodstream infections (HO-BSI) with the goal to expand prevention opportunities and further improve patient safety.^49^ Hospital-onset bacteremia broadens our prevention perspective to all HO-BSI and not only the small subset (<10%) of HO-BSI that are CLABSI. As with the CLABSI definition described above, the impact of adopting more sensitive non–culture-based tests on HO-BSI could be ameliorated by preferentially using the results of blood cultures obtained in a specific time interval around the non–culture-based test.

New technologies will likely improve diagnostic precision. Implementing new technology with consideration of diagnostic stewardship is important. Public health agencies that track laboratory-defined HAIs must be proactive to adjust to new technology. Creation of diagnostic stewardship and/or antibiotic stewardship teams or programs will be needed to monitor and implement these new diagnostics.

In conclusion, HAI surveillance is performed to detect preventable adverse events and to permit accurate comparisons between hospitals and track trends over time. The relationship between diagnostic testing and HAI rates has incentivized changes in testing, which may not always be aligned with diagnostic stewardship principles. A growing body of literature supports appropriate testing to improve patient care through less misclassification of colonization and fewer reported HAIs. Blanket reductions or changes to testing practices without diagnostic stewardship could result in unintended adverse consequences. By focusing on a patient-centered approach, diagnostic stewardship aligns with patient quality and safety goals to achieve desired outcomes. To encourage optimization of diagnostic testing that improves patient outcomes, HAI definitions need to consider additional clinical criteria such as the decision to start treatment for the infection. In addition, process measures need to be put in place to track inappropriate testing, and support for infrastructure and staffing to monitor diagnostic stewardship efforts need to be in place to ensure sustainability of diagnostic stewardship interventions and maintain focus on patient safety.
